# The Role of Duplex Ultrasound and Flow/Pressure Ratio in Predicting Invasive Treatment for High Venous Pressure in Patients Undergoing Hemodialysis

**DOI:** 10.3390/medicina60122055

**Published:** 2024-12-13

**Authors:** Foong-Fah Leong, Wen-Chin Lee, Hwee-Yeong Ng, Po-Yen Kuo, Chien-Te Lee, Chung-Ming Fu

**Affiliations:** 1 Division of Nephrology, Department of Internal Medicine, Kaohsiung Chang Gung Memorial Hospital, Chang Gung University College of Medicine, Kaohsiung 833401, Taiwan; 2 Division of Nephrology, Department of Internal Medicine, Kaohsiung Municipal Feng-Shan Hospital (Under Management of Chang Gung Medical Foundation), Kaohsiung 83062, Taiwan

**Keywords:** hemodialysis, high venous pressure, duplex ultrasound, vascular access, flow/pressure ratio

## Abstract

*Background and Objectives*: Vascular access complications, particularly high venous pressure, pose significant challenges for hemodialysis patients undergoing hemodialysis. Limited research has focused on identifying predictive factors for invasive treatment. This study aimed to identify patients who might benefit from frequent monitoring and conservative management based on duplex ultrasound (DUS) evaluation. *Materials and Methods:* This retrospective study included 72 hemodialysis patients with high venous pressure who underwent DUS. Patients were divided into conservative (*n* = 26) and invasive treatment groups (*n* = 46). Key factors such as flow/pressure ratio, blood flow, and venous pressure were analyzed. Logistic regression was used to identify risk factors for invasive treatment, while receiver operating characteristic (ROC) analysis was performed to establish the optimal cutoff for the flow/pressure ratio. *Results:* Three months after the DUS, 3.85% of the conservative group experienced access failure, compared to 71.7% in the invasive group. The flow/pressure ratio was significantly lower in the invasive group (1.28 ± 0.26 vs. 1.47 ± 0.23, *p* < 0.05). A higher flow/pressure ratio (OR: 0.063, 95% CI: 0.004–0.932, *p* = 0.044) and the presence of tortuous veins (OR: 0.080, 95% CI: 0.007–0.897, *p* = 0.0405) were associated with a lower risk of invasive treatment. ROC analysis showed a flow/pressure ratio cutoff of 1.38 (AUC: 0.706, *p* = 0.004). *Conclusions:* Duplex ultrasound plays a crucial role in evaluating arterio-venous access in patients with high venous pressure. It provides a non-invasive assessment of vascular complications, helping avoid unnecessary invasive procedures. The flow/pressure ratio is significantly associated with the risk of invasive treatment, providing a valuable threshold for assessing risk and guiding clinical decision-making to optimize treatment strategies.

## 1. Introduction

Vascular access is essential for effective hemodialysis (HD) and plays a vital role in the survival and quality of life of patients undergoing maintenance HD [1]. Providing appropriate vascular access for HD remains one of the most challenging concerns. The vascular access may be a dialysis central venous catheter or, preferably, a permanent HD arteriovenous (AV) access, such as an AV graft (AVG) or a native AV fistula (AVF) [2].

A properly functioning AV access is characterized by low resistance and adequate venous outflow [3]. However, various complications may alter these characteristics, and high venous pressure is an important concern that is often underestimated in clinical practice [4,5]. Although reduced blood flow often prompts immediate referral to cardiovascular surgery or interventional radiology, the clinical consequences of high venous pressure are often overlooked [4,6].

High venous pressure in HD access can result from several factors, including venous stenosis, thrombosis, central venous obstruction, high-flow fistula, or valvular incompetence in the outflow vein [7,8]. It can lead to complications such as access site bleeding, prolonged hemostasis, arm swelling, reduced dialysis efficiency, and, if untreated, thrombosis and access failure [9]. Early detection and management of high venous pressure is critical to maintaining functional vascular access. Traditionally, contrast venography has been the gold standard for vascular assessment. However, it has limitations, including invasiveness, risk of allergic reactions, and potential for contrast-induced nephropathy [10]. In recent years, duplex ultrasound (DUS) has become an important tool in assessing AV access. It provides a noninvasive method of evaluating the vascular systems without exposure to radiation or nephrotoxic contrast agents [11]. Importantly, DUS can be used for vessel mapping to determine appropriate cannulation sites [12,13]. As demonstrated in our previous study, ultrasound-guided vessel mapping can significantly improve AVF creation and maturation success rates [14]. Given the potential of DUS in assessing vascular access, it is important to explore its role in managing high venous pressure. This study focuses on understanding the causes of high venous pressure and evaluating how DUS can guide treatment decisions. Our goal was to use DUS to identify the underlying causes and develop appropriate treatment strategies based on the findings.

## 2. Materials and Methods

### 2.1. Study Design and Participants

This retrospective cohort study recruited patients receiving maintenance HD from the HD center of the Kaohsiung Chang Gung Memorial Hospital between January 2017 and December 2019. At our facility, a dedicated team of nephrologists conducts regular assessments of vascular access for patients undergoing long-term hemodialysis with AVFs or AVGs, using ultrasound when necessary to provide detailed evaluations of the vascular access. The objective was to assess the role of DUS in identifying the etiology of high venous pressure and to determine treatment strategies for patients based on DUS findings.

### 2.2. Inclusion and Exclusion Criteria

Patients who were identified by medical staff to have high venous pressure during HD (defined by venous pressures greater than 150 mmHg (using a 16G gauge needle) at a blood flow of 200 mL/min for at least three consecutive sessions) and received DUS examination were included in this study [15]. Exclusion criteria included patients who did not receive regular HD for more than 12 months at the facility or those lost to follow-up after the DUS evaluation.

### 2.3. Data Collection and Definition

Demographic data, including age, gender, and dialysis vintage, was collected. Laboratory evaluations including serum sodium, potassium, phosphorus, calcium, intact parathyroid hormone (iPTH), albumin, uric acid, blood urine nitrogen (BUN), creatinine, hemoglobin (Hgb), platelet (Plt), ferritin, transferrin Saturation (TSAT), high sensitive C-reactive protein (HS-CRP), Kt/V (K: clearance of urea, t: time, V: volume of distribution of urea) and URR (Urea Reduction Ratio) before receiving DUS, were also collected. Comorbidities, including hypertension, diabetes mellitus, coronary artery disease, peripheral arterial occlusive disease, heart failure, valvular heart disease, and atrial fibrillation, were retrospectively recorded via review of electronic medical records. Coronary artery disease and peripheral arterial occlusive diseases were confirmed by examination of angiograms. Hypertension was defined as taking anti-hypertensive medications or systolic blood pressure >140 mmhg. Heart failure was defined based on a documented diagnosis in the medical record or a cardiac echocardiogram indicating an ejection fraction (EF) of less than 40%. Valvular heart disease was defined based on a documented diagnosis in the medical record or a cardiac echocardiogram indicating moderate to severe aortic stenosis (AS), aortic regurgitation (AR), mitral stenosis (MS), or mitral regurgitation (MR). Diabetes mellitus was defined as taking oral antidiabetic agents, insulin therapy, or a glycohemoglobin level of >6.5%. Medication exposure to antiplatelet or anticoagulant therapy was also recorded.

Characteristics of the AV access, including location and type, were analyzed. Blood flow and venous pressure during HD were also recorded. The ratio between blood flow and venous pressure was calculated. The etiology of venous hypertension was analyzed and recorded. Stenosis was defined by a vascular lumen reduction of >50% and a peak systolic velocity (PSV) ratio across the stenotic area of >2.0. Occlusion was defined as the collapse of the graft wall, and no duplex signal was detected from the vessel [16]. A small drainage vein was defined as a diameter <6 mm, and high blood flow was defined as blood flow of >1.5 L/min under DUS [17,18].

### 2.4. Procedures

All enrolled patients underwent DUS evaluation using a Toshiba Xario XG machine with a high-frequency (7–15 MHz) linear probe. The ultrasound evaluation focused on the feeding artery, arteriovenous anastomosis, and outflow vein. The ultrasound operator screened the entire drainage system and provided a ‘vascular map report’ for all patients who underwent the examination. Vascular parameters such as resistance index (RI), pulsatility index (PI), and maximum drainage vein diameter were measured. The vascular map will indicate potential causes of elevated venous pressure and problematic areas. Simultaneously, the ultrasound operator will identify an alternative site for cannulation that meets the criteria of a minimum of at least 5 cm between two cannulation needles, vessel diameter >6 mm, and vessel depth <6 mm [19].

**Figure 1:**
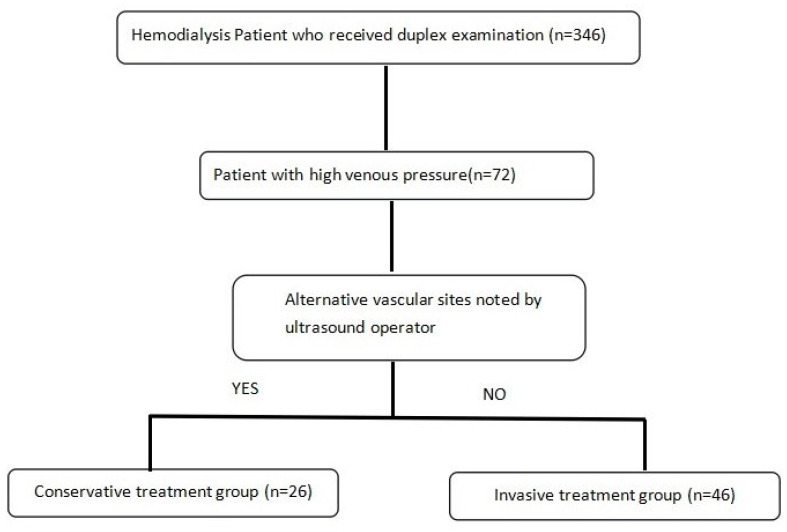
Flowchart of patient enrollment and treatment pathways.

Based on the DUS findings, patients were stratified into two groups: patients with an alternative cannulation site were assigned to the conservative treatment group, and those with no adequate cannulation site were assigned to the invasive treatment group. Patients classified to the invasive treatment group were referred to the Division of Cardiovascular Surgery or Cardiology for further management (Figure 1).

All patients were followed up for 3 months after the examination to observe whether they received invasive treatment [e.g., percutaneous transluminal angioplasty (PTA), new AV access creation, endarterectomy of AV access, superficialization of AV access, and tunnel dialysis catheter (TDC) insertion] by the Division of Cardiovascular Surgery or Cardiolog. 

### 2.5. Statistical Analysis

Continuous variables were tested for normality using the Shapiro–Wilk test. Variables with normal distributions are presented as mean ± standard deviation (SD) and compared using a two-sample *t*-test. For non-normally distributed variables, the Mann–Whitney U test was applied, and the results are expressed as medians with interquartile ranges (IQR). Categorical variables were analyzed using Chi-square tests. Logistic regression analysis was performed to identify independent risk factors associated with the need for invasive treatment. Receiver operating characteristic (ROC) curve analysis was conducted to determine the optimal flow/pressure ratio cutoff for predicting invasive treatment using the Youden Index method, which identifies the threshold that maximizes the sum of sensitivity and specificity. A *p*-value of <0.05 was considered statistically significant. The statistical analysis was conducted using SAS, version 9.4.

## 3. Results

### 3.1. Demographics and Hemodialysis Parameters of Patients with High Venous Pressure 

Three hundred and forty-six stable patients undergoing HD received DUS during the study period, and seventy-two patients received the examination due to high venous pressure. Among the patients who had high venous pressure, 26 patients were classified in the conservative treatment group, and 46 patients were classified as requiring invasive strategies according to the protocol. The percentage of patients with a native arteriovenous fistula (AVF) was significantly higher in the conservative group compared to the invasive group (96.2% vs. 78.3%, *p* < 0.05). Additionally, significant differences in HD blood flow, venous pressure, and the flow/pressure ratio were observed between the groups, with the invasive treatment group revealing significantly lower values (246.4 ± 29.2 vs. 264.3 ± 22.3 mL/min for blood flow, *p* < 0.05; 183.4 ± 35.2 vs. 197.3 ± 30.4 mmHg for venous pressure, *p* < 0.05; 1.28 ± 0.26 vs. 1.47 ± 0.23 for flow/pressure ratio, *p* < 0.05). However, no notable differences were observed in laboratory findings between the groups, except for potassium levels. Potassium levels in the invasive treatment group were significantly higher than those in the conservative treatment group (4.8 ± 0.7 vs. 4.5 ± 0.4, *p* < 0.05), suggesting that clearance may be poorer in the invasive group due to the critical condition of the AV access (Table 1).

### 3.2. Etiologies of High Venous Pressure and Clinical Outcomes

The etiologies of high venous pressure in our patients included drainage vein stenosis (42%), drainage vein occlusion (13%), central vein stenosis (8%), tortuous drainage veins (11%), small drainage veins (8%), and high fistula blood flow (13%). Among these, drainage vein stenosis was most strongly associated with the need for invasive treatment (80% of patients in the invasive group had stenosis compared to only 20% in the conservative group). Conversely, patients with tortuous veins were more likely to be managed conservatively (75% of these patients were in the conservative treatment group, *p* < 0.05). Regarding clinical outcomes, the data suggest that the decision to pursue conservative treatment was validated by a low access failure rate in this group. Specifically, three months after the initial decision, only 3.85% of patients in the conservative treatment group experienced access failure, compared to a 71.7% intervention rate in the invasive treatment group. (Table 2).

### 3.3. Predictors and ROC Curve Analysis of Invasive Treatment

**Figure 2:**
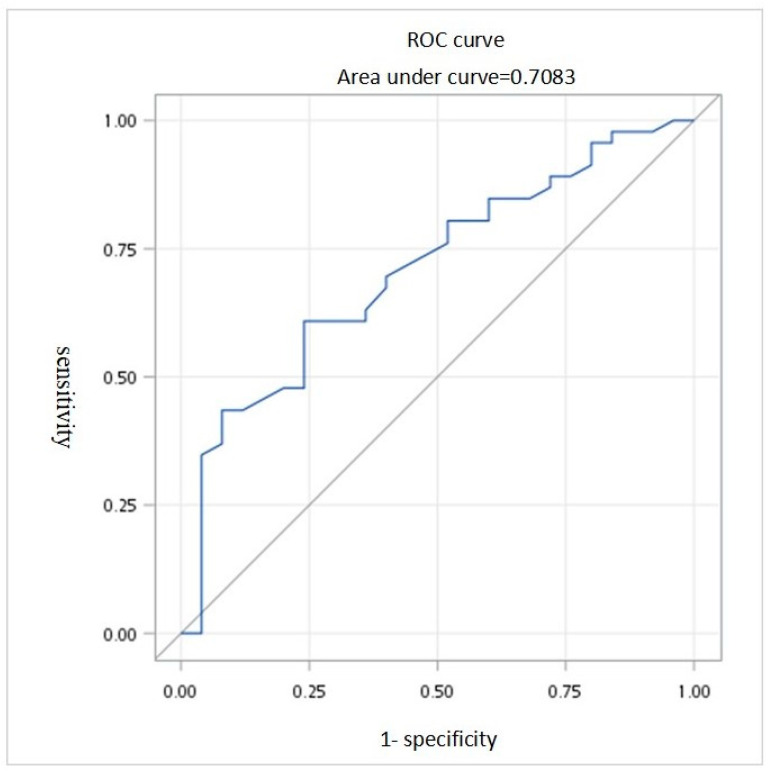
Receiver operating characteristic (ROC) curve of flow/pressure ratio. The flow/pressure ratio threshold of 1.38 has a sensitivity of 0.76, specificity of 0.61, and area under the curve (AUC) of 0.706 (95% confidence interval [CI],0.581–0.830, *p* = 0.004).

The parameters that differed significantly between the conservative and invasive treatment groups, including AV access type, drainage vein stenosis, tortuous drainage vein, potassium level, high fistula blood flow, and flow/pressure ratio, were used for Logistic regression analysis. The model did not include the AV access flow rate and venous pressure before the examination due to collinearity with the flow/pressure ratio. The analysis demonstrated that a higher flow/pressure ratio (OR: 0.063, 95% CI: 0.004–0.932, *p* = 0.044) and the presence of tortuous veins (OR: 0.080, 95% CI: 0.007–0.897, *p* = 0.0405) were associated with a significantly lower risk of requiring invasive treatment. In contrast, factors such as stenosis, potassium levels, and high blood flow fistula were not significantly associated with the risk of invasive intervention. These results suggest that both a higher flow/pressure ratio and the presence of tortuous drainage veins serve as protective factors, lowering the likelihood of requiring invasive treatment. (Table 3) ROC curve analyses were performed to assess the flow/pressure ratio (Figure 2), with an AUC of 0.708 (95% confidence interval [CI], 0.583–0.834, *p* = 0.001) and a cut-off point of 1.38. 

## 4. Discussion

The present study highlights the significant role of DUS in managing patients with high venous pressure in HD. Several significant findings have emerged from our cohort. First, we noted that drainage vein stenosis is the most common cause of elevated venous pressure and the primary indicator for invasive treatment. Some patients with high venous pressure caused by tortuous drainage veins may be treated without invasive therapy. Second, compared with patients with native AVF who develop high venous pressure, patients with AVG with high venous pressure are more likely to require invasive treatment. Third, the flow/pressure ratio was lower in patients who required invasive treatment, making it a critical risk factor for identifying individuals likely to need invasive procedures. In our cohort, stenosis (42%) was the most common cause of high venous pressure, and percutaneous transluminal angioplasty (PTA) was the most frequently used treatment (80.4%) when invasive intervention was required. Similar findings have been reported in previous studies [5,20]. However, by using DUS prior to referral for invasive therapy, additional causes of high venous pressure, such as drainage vein thrombosis, central vein stenosis, tortuous drainage veins, small drainage veins, and high fistula blood flow in AV access, were identified in our study. With the aid of a vascular mapping report using DUS, 36.11% of patients with high venous pressure were successfully managed without the need for invasive treatment. Native AVFs are the preferred choice for patients undergoing HD due to their longer lifespan and lower need for interventions compared to arteriovenous grafts (AVGs) [21,22]. In our study, 84.93% of patients used AVFs, while the remaining used AVGs for HD. Patients with AVGs were more likely to require invasive treatment than those with AVFs. This is likely due to the higher risk of complications, such as early thrombosis, inadequate maturation, and increased susceptibility to venous stenosis, which typically result in poorer outcomes and a greater need for surgical intervention in patients with AVG [23,24,25]. Additionally, the difficulty in identifying alternative cannulation sites with DUS in patients with AVGs may further complicate their management and contribute to the increased referral for invasive treatments.

Early correction of abnormalities in Polytetrafluoroethylene (PTFE) grafts and in AV fistulas could prolong access life [26]. Several techniques have been utilized for the early detection of abnormal vascular events in AV access [5,27,28], with one of the most significant being blood flow monitoring. Regular blood flow monitoring through an AVF or graft (AVG) is crucial, as a significant drop in flow volume often signals potential stenosis [29]. However, one of the key findings in our study is the critical role of the flow/pressure ratio as a predictor for invasive treatment. Patients who required invasive interventions consistently had lower flow/pressure ratios, highlighting its potential as an early marker of vascular complications. This reduction in the flow/pressure ratio can be attributed to the clinical practice where nursing staff, upon detecting venous hypertension, reduce blood flow to complete the dialysis session. A lower ratio indicates that more drastic reductions in flow are needed, signaling a more severe underlying vascular issue that requires correction.

Our study demonstrates that the flow/pressure ratio is a powerful indicator directly associated with the likelihood of requiring invasive treatment. Specifically, ROC analysis identified a cut-off value of 1.38, with a sensitivity of 0.76, a specificity of 0.61, and an AUC of 0.708 (95% CI: 0.583–0.834; *p* = 0.001). When the flow/pressure ratio drops to this threshold, it becomes a clear signal for early referral and potential intervention. This finding highlights the importance of incorporating flow/pressure ratio monitoring into routine evaluations to more effectively identify patients at risk for severe vascular events and to guide timely interventions.

While the present study provides promising findings, several limitations should be acknowledged. First, the retrospective design may introduce bias, as the data collection relied on existing medical records, which could affect the accuracy and consistency of the results. Second, the relatively small sample size (*n* = 72) may limit the generalizability of the findings to a broader population of patients undergoing hemodialysis (HD). Third, the classification of patients into two groups involved a certain degree of subjectivity, which may have influenced the results. Despite these limitations, the study identifies the flow/pressure ratio as a key predictor for invasive treatment, offering clinicians practical and actionable insights. This novel quantitative metric has the potential to guide early interventions, improve patient outcomes, and reduce complications. Furthermore, the study highlights the clinical utility of using this ratio to assess the severity of vascular complications in patients with high venous pressure, even within the constraints of a relatively small sample size. Finally, the follow-up period of this study was limited to three months, which prevented an assessment of long-term outcomes. As a result, the durability of the interventions and the long-term effectiveness of conservative management strategies remain uncertain. 

Future studies with extended follow-up periods and a broader range of contributing factors are necessary to better understand the chronic implications of managing high venous pressure in patients undergoing HD.

## 5. Conclusions

DUS is highly valuable for investigating the underlying causes of high venous pressure in patients undergoing HD. It can help avoid unnecessary invasive procedures by comprehensively evaluating vascular access. Additionally, our findings further highlight the significance of the flow/pressure ratio as a critical risk factor in determining the need for invasive treatment. A lower flow/pressure ratio is associated with more severe vascular conditions and can guide timely interventions. Given its effectiveness in assessing high venous pressure and predicting the severity of vascular complications, we recommend that DUS be routinely performed in all patients before considering invasive interventions. This approach facilitates more accurate diagnoses and supports informed decision-making in the management of high venous pressure.

## Figures and Tables

**Table 1:** Demographics of hemodialysis patients with high venous pressure.

Parameters	N = 72	Conservative (N = 26)	Invasive (N = 46)
Age (years)	62.5 (54.0~68.0)	62.0 (52.0~67.0)	62.0 (56.0~68.0)
Sex (n, %)			
Male (n, %)	33 (45.8)	11 (42.3)	22 (47.8)
Female (n, %)	39 (54.17)	15 (57.7)	24 (52.2)
Vintage (years)	4.2 (1.8~9.7)	3.7 (1.8~6.3)	5.7 (1.9~11.8)
History of PAOD (n, %)	5 (6.9)	3 (11.5)	2 (4.3)
History of CAD (n, %)	16 (22.2)	9 (34.6)	7 (15.2)
History of HTN (n, %)	60 (83.3)	23 (88.5)	37 (80.4)
History of DM (n, %)	36 (50.0)	16 (61.5)	20 (43.5)
History of Af (n, %)	4 (5.6)	1 (3.8)	3 (6.5)
History of Hf (n, %)	9 (12.5)	5 (19.2)	4 (8.7)
History of Vd (n, %)	17 (23.6)	6 (23.08)	11 (23.9)
Use of anti-platelet drugs or Anti-coagulants (n, %)	21 (29.2)	10 (38.5)	11 (23.9)
AV access characteristics			
AV access side			
Left (n, %)	62 (86.1)	24 (92.3)	38 (82.6)
Right (n, %)	10 (13.9)	2 (7.7)	8 (17.4)
Type			
Graft (n, %)	11 (15.3)	1 (3.8)	10 (21.7) *
Duration of AV access (years)	2.3 (1.0~5.3)	2.1 (1.0~5.0)	2.7 (1.0~5.8)
Laboratory data			
Albumin (g/dL)	3.9 ± 0.3	4.0 ± 0.3	3.9 ± 0.3
BUN (mg/dL)	69.0 (58.0~80.0)	68.0 (54.5~78.0)	69.5 (62.0~80.0)
Cr (mg/dL)	10.6 ± 2.5	10.6 ± 2.6	10.6 ± 2.5
Na (mEq/L)	136.0 (133.0~139.0)	137.0 (133.0~139.0)	136.0 (133.0~138.0)
K (mEq/L)	4.7 ± 0.6	4.5 ± 0.4	4.8 ± 0.7 *
Ca (mg/dL)	9.3 ± 0.9	9.4 ± 1.1	9.2 ± 0.8
P (mg/dL)	5.2 ± 1.3	5.5 ± 1.0	5.0 ± 1.5
Hgb (g/dL)	10.7 (9.9~11.1)	10.8 (9.7~11.2)	10.7 (9.9~11.1)
Plt (1000/uL)	186.0 ± 60.8	193.5 ± 63.6	181.8 ± 59.6
Hs-CRP (mg/L)	2.0 (1.0~5.0)	3.5 (1.0~5.0)	2.0 (1.0~4.0)
iPTH (pg/mL)	253.0 (102.0~413.0)	216.0 (78.0~388.0)	268.0 (108.0~413.0)
Ferritin (ng/mL)	379.5 (162.0~635.5)	406.0 (157.0~635.0)	365.5 (175.0~657.0)
TSAT (%)	28.5 (22.0~34.5)	27.0 (19.0~33.0)	29.0 (24.0~37.0)
Kt/V	1.4 ± 0.2	1.3 ± 0.2	1.4 ± 0.2
URR	0.7 ± 0.1	0.7 ± 0.1	0.7 ± 0.1
Parameter during hemodialysis			
Blood flow rate (mL/min)	250.0 (245.0~277.0)	258.0 (250.0~280.0)	250.0 (228.0~253.0)
Venous pressure(mmhg)	188.5 (165.5~210.0)	168.5 (161.0~200.0)	193.5 (168.0~217.0)
Flow/pressure ratio	1.35 ± 0.26	1.47 ± 0.23	1.28 ± 0.26 *

Abbreviations: PAOD = Peripheral Arterial Occlusive Disorder, CAD = Coronary Artery Disease, HTN = Hypertension, DM = Diabetes Mellitus, Af = Atrial Fibrillation, Hf = Heart failure, Vd = valvular diseases, AV = Arteriovenous, BUN = Blood Urea Nitrogen, Cr = Creatinine, Na = Sodium, Ca = Calcium, P = Phosphorus, Hgb = Hemoglobin, Plt = platelet, Hs-CRP = High-Sensitivity C-Reactive Protein, iPTH = Intact Parathyroid Hormone, TSAT = Transferrin Saturation, Kt/V = K: clearance of urea, t = time, V = volume of distribution of urea, URR = Urea Reduction Ratio, * *p* < 0.05.

**Table 2:** Etiologies of venous hypertension and patency rate of AV access.

	Total (N = 72)	Conservative (N = 26)	Invasive (N = 46)
Etiology of high venous pressure			
Drainage vein stenosis (n, %)	30 (42)	6 (23.1)	24 (52.2) *
Drainage vein occlusion (n, %)	13 (18)	6 (23.1)	7 (15.2)
Central vein stenosis (n, %)	6 (8)	0 (0)	6 (13)
Tortuous drainage vein (n, %)	8 (11)	6 (23.1)	2 (4.3) *
Small drainage vein (n, %)	6 (8)	2 (7.7)	4 (8.7)
High fistula blood flow (n, %)	9 (13)	6 (23.1)	3 (6.5) *
Invasive treatment after 3 months (n, %)	34(47.2%)	1(3.85%)	33(71.7%) *

Abbreviations: * = *p* < 0.05.

**Table 3:** Logistic regression: odds ratios for the risk of receiving invasive treatment.

	Odd Ratio	95% Confidence Interval	*p* Value
Flow/pressure ratio	0.063	0.004~0.932	0.0443 *
Tortuous drainage vein	0.080	0.007~0.897	0.0405 *
Drainage vein Stenosis	1.468	0.348–6.199	NS
High fistula blood flow	0.264	0.031–2.247	NS
K (potassium level)	1.146	0.383–3.427	NS
Type (native versus graft)	0.094	0.006–1.585	NS

Abbreviations: * = *p* < 0.05; NS = no significant.

## Data Availability

Data are contained within the article.
